# FOBism Unveiled: Quantifying Assimilative Racism within Asians in the United States

**DOI:** 10.3390/ejihpe14100184

**Published:** 2024-10-16

**Authors:** Kenneth T. Wang, Seong-Hyeon Kim, Juliet K. Wang, Katelyn J. Wang, Helen H. Jun, Daniel D. Lee

**Affiliations:** 1 Fuller Theological Seminary, School of Psychology & Marriage and Family Therapy, Pasadena, CA 91101, USA; shkim@fuller.edu (S.-H.K.); jun.haein@gmail.com (H.H.J.); 2 School of Film and Television, Loyola Marymount University, Los Angeles, CA 90045, USA; 3 Department of Information and Computer Science, University of California, Irvine, CA 92697, USA; kateljw1@uci.edu; 4 Fuller Theological Seminary, School of Mission & Theology, Pasadena, CA 91182, USA; danieldlee@fuller.edu

**Keywords:** Asian American, internalized racism, FOB, scale development, psychometric evaluation

## Abstract

FOB (fresh-off-the-boat) is a term used to refer to unassimilated immigrants or sojourners, which has created a divide within the Asian community. In this study, we coined the term FOBism, a form of internalized racism (or appropriated racial oppression) that intersects with assimilation, and we developed a measure of FOBism. We created a 14-item, 3-factor FOBism Scale and evaluated its psychometric properties among a sample of 296 Asians in the United States. Exploratory structural equation modeling (ESEM) was utilized to select items and evaluate the factorial validity. Results yielded a strong factor structure, internal consistency reliability, and construct validity. Construct validity was demonstrated through FOBism scores’ positive correlations with measures of within-group discrimination and internalized racism, and negative associations with an Asian cultural orientation. The FOBism Scale is a promising measure that could be used as an assessment tool and to raise awareness of the phenomenon.

## 1. Introduction

Fresh-off-the-boat, or FOB, is a term used to describe someone who has recently immigrated and is unacculturated to the new country [1]. Though some have reclaimed this term, as evidenced by a popular Asian American sitcom naming itself unapologetically as *Fresh off the Boat* [2], FOB is generally considered derogatory. Stereotypical markers of Asians considered FOBs include using a foreign language, speaking English with an accent, preferring to eat Asian food over American food, and socializing more with other unassimilated Asians [1]. Researchers of this study have coined the term FOBism, which can be elementally defined as discrimination against characteristics or people who are considered FOBs and is a form of internalized racism (or appropriated racial oppression) that intersects with assimilationism, nativism, and orientalism. In the present study, we developed a scale to measure this pervasive yet often unspoken construct among Asians in the United States and to raise awareness of this intra-community bias.

The concept of the FOB is an example of how Asians become a victim of the White racial frame [3], which is when one takes a White vantage point by valuing White people and their culture, while devaluing other racial groups and their culture [4]. Liu and colleagues [5] argue that individuals in the process of assimilation are not only encouraged to adopt dominant cultural values seen as “accessible to everyone”, such as individualism and self-reliance, but also pushed to avoid racial discourse to minimize White fragility and distress surrounding racism, racial trauma, and microaggressions. Since assimilation can foster the White racial frame, which promotes silence around racial issues [3], it becomes even more crucial to elucidate the harmful processes by which concepts such as FOB interact with assimilation pressure, social identity, nativism, geopolitical tension, and the perpetual foreigner stereotype.

### 1.1. Conceptualizing FOBism from the Acculturation Perspective

As the concept of FOB has to do with culture and not merely race, it is necessary to define terms related to acculturation. Berry [6] defines psychological acculturation as the way individuals change upon contact with a new culture, and he uses a two-axis model to plot ways this process can manifest. The first axis measures how affiliated an individual is with their original culture, and the other measures how affiliated they are with the new culture. This model creates four quadrants of acculturation strategies: assimilation (rejection of original culture and acceptance of new culture), separation (acceptance of original culture and rejection of new culture), integration (acceptance of both original and new culture), and marginalization (rejection of both original and new culture). FOBism is marked by both a negative attitude toward the original culture and a positive attitude toward the new culture, so it aligns with an assimilation strategy, is most strongly opposed to separation, and is partly opposed to both integration and marginalization. Research suggests that the integration strategy has the best psychological and sociocultural outcomes, and that most immigrants prefer this strategy, but it can differ among groups [6]. Notably, in Berry et al.’s study [7], Vietnamese immigrants preferred assimilation nearly as much as integration, as opposed to a general sample, which greatly preferred integration. It is also important to recognize that the immigrant’s ideal strategy may not be actualized, and the attitudes of the larger, dominant society have a role in determining the acculturation strategy [6]. For example, the societal attitudes of the melting pot or exclusion both push immigrants to reject their original culture and adopt the assimilation or marginalization strategies, respectively. Experiences of discrimination also play a role in strategy adoption, and research suggests that more discrimination pushes immigrants toward separation, while less discrimination pushes immigrants toward assimilation or integration [8]. Asian Americans being situated as a model minority with less overt discrimination may be relevant here. Finally, it is also necessary to employ terms to describe having competency with one’s original culture and having competency with a new culture, and for this we will use Yun Young Kim’s [9] definitions of enculturated and acculturated, respectively. Note that “acculturated”, when used this way, is describing an individual’s competency with a new culture, with no implication of their competency with their original culture, and this should not be confused with Berry’s [6] broader use of “acculturation”, meaning the process of change upon contact with a new culture.

### 1.2. Defining FOBism as a Form of Internalized Racism

In the present study, we define FOBism as prejudice, discrimination, or antagonism directed against people of one’s own racial group who are less assimilated and/or against unassimilated characteristics in oneself. Asian reaction against non-assimilation through FOBism is a form of internalized racism—as both concepts include a marginalized racial group adopting the negative views that the oppressive group has toward them [10]. More specifically, under FOBism, Asians take both the oppressor’s xenophobia and perpetual foreigner stereotype and internalize them, directing them not only against unassimilated Asians through disassociation, de-identification, and other intra-ethnic othering strategies [4], but also toward themselves, by forcibly regulating their own behaviors and interests to avoid being perceived as a FOB [11]. Like general internalized racism, FOBism is also conceptualized to have three orientations [12]: interpersonal (FOBism toward others, e.g., looking down at others for speaking an Asian language in public), intrapersonal (FOBism toward oneself, e.g., avoiding speaking an Asian language in public), and institutional/societal (FOBism normalized in society, e.g., one’s primary language being an Asian one is seen as un-American).

### 1.3. FOBism from the Cultural Distancing Perspective

It is important to note that FOBism is not equivalent to internalized racism but is a more specific subset of attitudes. While internalized racism is based on race regardless of assimilation levels, FOBism is due to both race and culture and is highly dependent on perceptions of assimilation levels. The importance of examining culture in regard to in-group discrimination can be elucidated through the lens of Tajfel and Turner’s [13] social identity theory, which examines attitudes, self-image, and interactions among and between low- and high-status groups. Social identity theory posits that the push for social change from the low-status group comes in two pathways: individual mobility and social creativity. Individual mobility involves an individual trying to distance themselves from their low-status group to join the high-status group, while social creativity is a category of in-group solutions, which try to reframe status comparisons in various ways. Weak barriers to movement between groups stimulate individual mobility, and strong barriers stimulate social creativity. Changing one’s culture has relatively weak barriers compared to changing one’s race (or external perception of it), so it follows that the individual mobility pathway would be more associated with the cultural dimension, as opposed to the racial one. This emphasizes the importance of studying cultural distancing instead of only racial distancing, which is what distinguishes FOBism from internalized racism. Furthermore, Tajfel and Turner theorized social creativity as having three branches: (a) comparing the groups on a different dimension, (b) changing the perception of an in-group attribute to be more positive, and (c) changing what out-group the in-group is comparing and competing with from a higher-status group to a lower-status one. FOBism may express itself through the final branch as assimilated Asians moving their comparison and competition from a higher-status White American group to a lower-status, unassimilated Asian group.

### 1.4. FOBism from the Communication Perspective

In the arena of communication, another lens to view FOBism is through Orbe’s [14] co-cultural theory, which examines how marginalized groups communicate with dominant ones. In this theory, there are nine communication orientations formed by two factors: preferred outcome (which can come in the categories of assimilation, accommodation, or separation) and communication approach (with categories of nonassertive, assertive, or aggressive). FOBism falls under the assimilation category in the preferred outcome factor and can be any communication approach. This results in three communication orientations of FOBism: nonassertive assimilation (which could involve censoring one’s original culture and passively blending in to the new culture), assertive assimilation (which could involve extensive preparation and overcompensation to fit in with the new culture), and aggressive assimilation (which could involve violent communication, such as ridiculing aspects of the original culture in the self and others, as well as distancing from co-cultural group members).

### 1.5. Historical Impact

Beyond theory, Asians have undergone a fraught history of racism in the United States that may contribute to the prevalence of FOBism. Even recently, the rise in hate crimes against Asian Americans during the COVID-19 pandemic makes it salient that Asian American belonging has always been conditional [15]. This belonging is useful for the United States because the model minority myth maintains racist systems under the guise of equality, proven by the supposed success of Asians in this country [16]. Every so often, however, the myth’s usefulness is outweighed by other factors, and anti-Asian sentiment turns from implicit to overt, and this change does not happen in a vacuum.

The Yellow Peril ideology developed in the late nineteenth century as a reaction to the exploitation of Chinese immigrants at lower wages, which forced out White workers [17]. This ideology sees the Eastern world as an existential menace to the West, and it is explicitly racial: it is not concerned with any specific nationality; it does not even describe a human—it characterizes Asians as either animals or evil sorcerers. Before the end of the century, the Western powers would encourage and employ Yellow Peril as a justification for colonialism in China [18].

The pattern of the state encouraging anti-Asian sentiment to further its interests abroad continues throughout history. In World War II, the United States used propaganda to dehumanize the Japanese (e.g., posters depicting Japanese people as rats), stoking not only anti-Japanese sentiment in relation to the war abroad, but anti-Japanese sentiment against Japanese Americans at home [19]. What is crucial is that anti-Japanese sentiment was not national, but ethnic. At home, civilians created “Jap Hunting Licenses”, while the state created Japanese incarceration camps [17,20].

Killing is a significant moral and psychological challenge, and the atrocities of war are hard to justify. The murder of a human being is a horror, but when the enemy is not seen as human, it becomes easier for the soldiers, and the state can manufacture the consent of the public. Therefore, dehumanization and othering of the enemy’s race often accompanies warfare [21]. The popularization of the dehumanizing slur “gook” during the Korean War, the hyper-sexualization of Asian women justifying their rape during the Vietnam War, and the viewing of Central, South, and Western Asians as subhuman terrorists post-9/11 and through the wars in Afghanistan and Iraq are examples [17,20,22].

This political strategy continues today as tensions between the US and China rise, as the latter grows into a stronger world power. US government officials, including the former president, weaponized the COVID-19 pandemic against China by scapegoating them, and characterized Asians as both diseased, savage bat eaters and evil, mad scientists who unleashed a biological weapon on the world [23,24]. The key is that anti-Asian sentiment in the US does not appear by chance but is tied—and has a history of being tied—to geopolitical affairs, and may only get worse as international tensions increase, and as the US prepares, and tries to manufacture consent, for possible war (economic, armed, or otherwise), with China. This is relevant because if these tensions continue to rise while anti-Asian racism increases, FOBism may also increase, as Asian Americans may attempt to distance themselves from their original culture in order to try to prevent themselves from being seen as foreign enemies aligned with China.

### 1.6. Perpectual Foreigner Stereotype

This perception of Asians as foreigners or recent immigrants is a longstanding stereotype known as the perpetual foreigner stereotype [25]. The undercurrent of this form of nativism is the idea that no matter how long or how many generations Asians have lived in this country, their race forever ties them to foreignness, and they will never be seen as true Americans. To illustrate this, the stereotype is present every time an Asian American hears, “Where are you from?” followed by, “Yeah, but where are you *really* from?”; or even more brutally, “Go back to where you came from”.

The situation of Japanese American incarceration mentioned earlier is a result of this stereotype. The US government removed Japanese Americans from their homes, citing fear of them betraying the United States to help Japan [26]. This action implies that the United States saw Japanese Americans not as Americans who were loyal to their country, but foreigners who were loyal to Japan, which indicates that being ethnically Japanese meant one was perceived as a perpetual foreigner. The situation of anti-Asian attacks during the COVID-19 pandemic may also be linked to the perpetual foreigner stereotype. China was scapegoated for the pandemic, but many of the Asian victims of the hate crimes were not ethnically Chinese [27], and many of those that were had never been to China. The perpetrators were not only viewing all Asians as a monolith, but also operating under the perpetual foreigner stereotype, as they were blaming Asians in the US for a disease they believed was caused by the Chinese government and Chinese nationals.

As a defensive coping response to the perpetual foreigner stereotype, some Asians try to prove themselves as the exception by deliberately presenting as White-normative American as possible: for instance, avoiding Asian foods, Asian fashion, and other Asian people, while embracing mainstream American culture [1]. This causes a divide to form between Asians who are Americanized and those who are not. For example, there is not only pressure among the Asian American community to assimilate, but also pressure to not assimilate too much. The derogatory term “Whitewashed”, meaning too assimilated to White culture, is evidence of this pressure [28]. This division within the Asian community in the United States is prevalent but has been largely under-addressed within research literature.

### 1.7. FOBism in the Context of Existing Literature

We also want to make clear that the purpose of addressing and dismantling FOBism is not to push Asians in the US toward adopting a more traditionally “Asian” cultural identity. We recognize that Asian American identity is conceptualized through hybridity, and in light of diaspora, it is diverse, dynamic, and heterogenous [29]. Therefore, it is necessary to resist the urge to solidify some rigid notion of an authentic Asian American identity. The purpose of combating FOBism is not to seek this rigidity, but to remove discriminatory pressures of forced assimilation and thereby allow individuals to freely form their own cultural identities.

Existing measures of Asian American internalized racism include the Internalized Racism in Asian Americans Scale (IRAAS) [30] and the Colonial Mentality Scale (CMS) [31]. They are both multi-dimensional, and the IRAAS includes three dimensions: Self-Negativity, Weakness Stereotypes, and Appearance Bias, whereas the CMS includes five dimensions: Within-Group Discrimination, Physical Characteristics, Colonial Debt, Cultural Shame and Embarrassment, and Internalized Cultural/Ethnic Inferiority. The creation of a FOBism scale is critical, as it addresses the often overlooked but widespread issue of intra-group bias directed toward members who are less assimilated within the community of Asians in the US [1,4,32]. In short, the FOBism Scale addresses the intersection between culture and race, while the other two internalized racism scales primarily regard only race.

### 1.8. The Current Study

In response to the call for more studies that examine racial experiences specific to various ethnic/racial groups [12], the key aim of this study is to develop a measure to assess FOBism among Asians in the United States. To establish construct validity, we examined FOBism’s relationship with other hypothetically associated constructs.

## 2. Materials and Methods

### 2.1. Participants

The participants were recruited through various methods, such as invitation emails sent to 178 international student offices and 72 Asian American centers at various universities in the US requesting their help to disseminate to their students, 17 direct invitation posts on social media pages centered around Asian American issues (e.g., Subtle Asian Traits, Asian Americans Advancing Justice–Los Angeles), and the utilization of a snowball sampling method. The study was approved by the IRB of the researchers’ institution and the online survey was conducted through Qualtrics. For every 10 valid completed surveys, the research team donated USD 5 to the Asian Mental Health Collective.

A total of 296 participants were included in this study sample after removing those who were ineligible, either due to incorrectly answering a validity-check question (*n* = 41) or indicating being under 18 years old (*n* = 2). All participants were Asians in the United States (200 women, 90 men, 5 nonbinary individuals, and 1 other). Mean age was 33.17 years (*SD* = 13.07). The majority only indicated Asian as their race (96%), whereas 4% noted being multi-racial. Roughly half (52%) of the individuals were native-born Americans, while the rest originated from various other nations. The list of birth countries outside the United States, arranged alphabetically, included Australia, Bahrain, Bangladesh, Burma, Canada, China, Guam, India, Indonesia, Ireland, Laos, Lebanon, Malaysia, Mongolia, Nepal, the Netherlands, the Philippines, Singapore, South Korea, Spain, Sri Lanka, Taiwan, Thailand, and Vietnam. In terms of participants’ generational status in the United States, 36% were first generation, 13% 1.5 generation, 37% second generation, and 14% were third generation and beyond. Approximately half were students (52%) and the other half working (42%) or other (6%). The participants resided in various states across the United States, with California (42%), Missouri (12%), and Kansas (10%) among the top-three locations. As for the percentage of Asians in their community, 44% of participants indicated less than 10% of Asians in their community, 35% of participants indicated Asian representation between 10 and 33%, 11% of participants indicated Asian representation between 33 and 50%, and 10% of participants indicated that more than half of their community was represented by Asians.

### 2.2. FOBism Scale Item Development

#### 2.2.1. Initial Development

The researchers of this study generated the initial items for the FOBism Scale after multiple iterative reviews and examinations on the literature on the psychology of Asians in the US and internalized racism, coupled with in-depth group discussions. Items were all originated and written by the research team following the structure of a tripartite framework—interpersonal, intrapersonal, and institutional—drawing on David and colleagues’ [12] orientations of internalized racism. We used the term societal to replace institutional to better reflect the context of FOBism.

The interpersonal category included items examining FOBism directed toward others. The intrapersonal items were focused on FOBism directed toward oneself. Finally, the institutional (societal) category of items was created to address FOBism normalized in society. Items were focused more on behavioral and attitudinal aspects. After careful review of items to ensure clarity, conciseness, readability, distinctiveness, and reflection of the FOBism Scale’s purpose, as suggested by Worthington and Whittaker [33], a total of 55 items were included in the initial item pool.

#### 2.2.2. Revise and Polish

The initial pool of 55 items was then reviewed by a team of 4 psychology doctoral students with familiarity with the psychology of Asians in the US. This second round of review focused on the clarity of items and the item–category match. Several items were revised to enhance clarity and consistency. A few items were deleted, while a few items were added, resulting in a total of 53 items.

#### 2.2.3. Expert Review

Three experts in the field of Asian American Psychology were then approached to evaluate the quality and representation of the items against the FOBism construct. The three experts consisted of an established Asian professor in psychology, an early-career Asian faculty member in psychology, and a dean of an Asian American Center. They were first asked to categorize the items as interpersonal, intrapersonal, and institutional (societal). Second, the experts were asked to indicate the extent to which each item reflected the definition of FOBism (relevancy) and whether there were aspects of the item that were unclear (clarity), and they were invited to edit the items, suggest additional items, and provide constructive feedback to improve the scale. The research team incorporated the feedback and made revisions accordingly. The item pool was further trimmed down to 9 items per category, resulting in a final FOBism pool of 27 items.

#### 2.2.4. FOBism Scale Item Pool

The FOBism Scale Item Pool includes 27 items that measure prejudice, discrimination, or antagonism directed externally against people who are less assimilated and/or internally against unassimilated characteristics in oneself. Each item is rated on a 6-point Likert scale (1 = strongly disagree, 2 = disagree, 3 = somewhat disagree, 4 = somewhat agree, 5 = agree, and 6 = strongly agree). Sample items include: “I look down on Asians who dress like foreigners” (interpersonal), “I avoid speaking an Asian language in public” (intrapersonal), and “Negative Asian stereotypes mostly stem from recent immigrants” (institutional/societal). The instructions to participants were as follows: “Below are some items about your views and attitudes regarding Asian people and cultures in the US. Please respond to the following items as honestly as possible”.

### 2.3. Other Measures for Construct Validity

#### 2.3.1. Colonial Mentality

The Colonial Mentality Scale (CMS) [31] measures a form of internalized oppression, and consists of five different subscales. The CMS was originally used to study Filipino immigrants, but for this study, we adapted it to the broader category of Asian immigrants and focused on within-group discrimination. This 11-item scale is rated on a Likert Scale ranging from 1 (strongly disagree) to 6 (strongly agree), with statements such as, “I generally do not like newly arrived (FOBs) Asian immigrants”. The within-group discrimination subscale has reliable internal consistency, with a Cronbach’s alpha of 0.89 [31].

#### 2.3.2. Internalized Racism

The Internalized Racism in Asian Americans Scale (IRAAS) [30] assesses the degree to which Asians possess negative feelings about their racial identity. The items are rated on a Likert-type scale from 1 (strongly disagree) to 6 (strongly agree). An example item is “It’s unfair that I was born Asian”. A bifactor model was found to have the best fit, indicating that the vast majority of reliable variance originated from a single source, suggesting the use of a total score.

#### 2.3.3. Foreigner Stereotype

Participants were assigned to answer one of the following two measures assessing how they have been micro-aggressively treated as foreigners. Due to the nature of different lived experiences, those who came to the United States after 13 years of age were presented with the Stress over the Perpetual Foreigner Stereotype Scale, which was more applicable to those who came to the United States at a later age because they were more likely to exhibit observable characteristics of immigrants or sojourners. For those who were born in the United States or came at age 12 or younger, they were presented with the Foreigner Objectification Scale, which was designed to assess being perceived as foreigners despite presenting as American as anyone else.

The Stress over the Perpetual Foreigner Stereotype Scale (SPFS) [34] was initially designed for Chinese American parents to measure the stress of being perceived as foreigners. It consists of five items, such as, “People criticize me for not speaking/writing English well”, which are rated on a Likert-type scale from 1 (not stressful) to 4 (very stressful), indicating the stress of those experiences. The scale proves to have strong internal consistency, with a Cronbach’s alpha of 0.90 for both Chinese American mothers and fathers [34].

The Foreigner Objectification Scale (FOS) [35] measures the degree to which ethnic minorities in the United States are perceived as perpetual foreigners from interpersonal interactions. Participants are asked to rate four items on a Likert-type scale from 0 (never) to 5 (five or more times), indicating the frequency of experiences, such as having “your American citizenship or residency questioned”.

#### 2.3.4. Acculturation

The Asian American Multidimensional Acculturation Scale (AAMAS) [36] assesses the extent to which Asian Americans identify with their culture of origin, Asian Americans and European Americans. For our study, we compiled 10 items from the factors of People, Culture, and Language, which were rated on a Likert-type scale from 1 (not very much) to 6 (very much). The People factor was assessed through four items, with statements such as “How much do you feel you have in common with” followed by “Asian Internationals”, “Asian Americans”, and “European Americans”. The Culture factor was assessed through two items, with statements such as “How much do you identify with” followed by “Your Asian Culture of Origin” and “White Mainstream American Culture”. The Language factor was assessed through four items, with statements such as “How often do you listen to music or watch movies/shows in” followed by “An Asian language” or “English”.

#### 2.3.5. Collective Self-Esteem

The Collective Self-Esteem Scale (CSES) [37] evaluates how a person’s social identity affects their concept of self. In this study, we focused on the Identity and Private subscales, which consider how an individual’s ethnic group reflects personal identity and how the individual feels about belonging to their ethnic group, respectively. The items are rated on a 7-point Likert-type scale ranging from 1 (strongly disagree) to 7 (strongly agree). A sample item is “The ethnic group I belong to is an important reflection of who I am”. The scale has acceptable internal reliability, with Cronbach’s alphas ranging from 0.71 to 0.86 for the Private and Identity subscales across three different studies [37].

#### 2.3.6. Depression

The Depression Anxiety Stress Scale (DASS) [38] was used to measure depression. Participants indicated how much each item applied to them in the past week on a scale from 0 (did not apply to me at all) to 3 (applied to me very much, or most of the time). A sample item reads, “I felt that I had nothing to look forward to”. With an Asian sample, the Cronbach’s alpha was 0.86 for the depression subscale [39].

#### 2.3.7. Social Desirability

The Five-Item Measure of Socially Desirable Response Set (SocDes) [40] examines the extent to which individuals attempt to present themselves in a more favorable, socially desirable light. The scale is ranked on a scale from 1 (definitely true) to 5 (definitely false) and includes statements such as “I am always courteous, even to people who are disagreeable”. The internal consistency reliability ranges from a Cronbach’s alpha of 0.66 to 0.75 in three different samples [40].

### 2.4. Study Hypotheses

Theoretically, higher FOBism levels are exhibited through a cultural orientation toward mainstream American norms. Thus, we anticipated FOBism to be positively correlated with identifying with European Americans and negatively correlated with identifying with Asian Americans or Asian internationals. We also anticipated FOBism to be positively correlated with acculturative behaviors toward mainstream American culture, as well as using English as one’s primary language.

In addition, FOBism is a form of internalized racism and colonial mentality [12]. Thus, we anticipated FOBism scores to be positively related to measures of within-group discrimination and internalized racism. Moreover, FOBism is a phenomenon resulting from decades of being perceived as foreigners in the United States [41]. We, therefore, anticipated a positive association between FOBism and discriminatory experiences of the perpetual foreigner stereotype and foreign objectification. As FOBism is associated with feeling inferior as an Asian person [1], we hypothesized FOBism to be negatively associated with collective self-esteem. Although we examined the relationship between FOBism and depression, this was exploratory in nature, and we did not have a specific hypothesis of the relationship between these two constructs. Lastly, we examined discriminant validity through FOBism’s association with social desirability; thus, we anticipate a nonsignificant correlation between these two constructs.

### 2.5. Statistical Analysis Plan

Exploratory structural equation modeling (ESEM) was conducted through MPlus version 7.11 to evaluate the factorial validity of the scale, through which the authors attempted to identify a subset of items best fitting the three-orientation framework behind the scale construction. ESEM combines the strengths and addresses the limitations inherent in exploratory factor analysis (EFA) and confirmatory factor analysis (CFA).

Since the three-factor structure is based on David and colleagues’ [12] three orientations of internalized racism, the process started directly with ESEM. Despite its wide popularity, CFA has been criticized by many for unrealistic restrictions imposed on the factor–indicator and indicator–indicator relationships [42]. For example, CFA, in general, imposes a simple structure that dictates indicators to load on only one factor, thus rarely allowing cross-loadings on the secondary factor, with mostly zero cross-loadings. Preventing cross-loadings results in potential poor fit as well as a serious issue of inflating inter-factor correlations [42,43]. The suppressed cross-loadings that are artificially fixed to zero in CFA tend to be funneled to factor correlations instead, and these exaggerated correlations are induced to blur the discriminant validity of the constructs measured by CFA factors. In addition, the EFA method is limited as a validation tool for the FOBism Scale primarily due to EFA’s assumption of a diagonal residual matrix, which precludes the inclusion of correlated residuals within its model [44]. The use of ESEM has been on the rise in the social sciences mainly to investigate the factorial validity and/or measurement invariance of psychological measures [45,46].

## 3. Results

### 3.1. Item Selection and Validation

For the current ESEM analysis on the FOBism Scale dataset, a 3-factor model on the 27 items, with 9 corresponding to each factor, was conducted. The target rotation matrix was specified to allow items to freely load on their primary factor, but their loading to non-primary factors was specified to approximate zero. Modification indices (MIs) were considered when searching for the best-fitting model, and items were carefully inspected through substantive or theoretical support.

Multiple ESEM models with varying sets of indicators were evaluated to identify a best set of items that represents FOBism well, both theoretically and parsimoniously. Considering the categorical nature of item responses, a robust weighted least squares estimator (WLSMV) was employed. ESEM models were evaluated using goodness-of-fit indices, such as the comparative fit index (CFI), the Tucker–Lewis index (TLI), and the root mean square error of approximation (RMSEA).

A series of five ESEM models with the same three factors were fit to a successively smaller set of items. The chi-square test of model fit and the goodness-of-fit indices for the five ESEM models are reported in Table 1. The first ESEM model with 27 items was fit to the item responses. Nine items were removed due to low communality (<0.30) and/or small loadings on the primary factor (<0.40). Three more items were removed in the second round due to small loadings on the primary factor (<0.40) and/or a large loading on a non-primary factor larger than 0.30 (i.e., non-ignorable cross-loadings). The third ESEM model with 15 items demonstrated a fit between reasonable and good. Modification indices were consulted for model improvement, which suggested a significant residual correlation between items #10 and #18. These two items share a common phrase in them, “Asians [living] in the US…” and both belong to Factor 3. The 15-item ESEM model with a correlated residual variance between items #10 and #18 showed a significantly improved model fit over the model without it (Δ*χ*^2^ (1) = 34.951, *p* < 0.001); however, item #10′s primary loading on Factor 3 dropped to below 0.4 in this model. In addition, the content overlap of the two items was deemed to support a removal of either. Thus, item #10 was removed from the analysis and a 14-item ESEM model was fit to the data. This final ESEM model showed a fit between ‘good’ and ‘excellent’ based on the fit indices. A close examination of the factors–indicators relationships and their substantive fit also supported the final 14-item FOBism Scale.

The factor loadings and the inter-factor correlations of the 14-item model are reported in Table 2. All factor loadings on the primary factor were larger than 0.50, and cross-loadings were less than 0.30. The three factors showed significant correlation between them (around *r* = 0.50, *p* < 0.001). If a CFA approach had been attempted rather than ESEM, these correlation coefficients would have been larger, thus blurring the discriminant validity of each factor.

Because these significant correlations between the three first-order factors implied a presence of a second-order factor explaining the covariance among the three first-order factors, we fitted a higher-order ESEM model, known as the ESEM-within-CFA (EWC) model [47]. The EWC model was simply a re-expression of the final 14-item ESEM model within a CFA framework, where the estimated factor loadings were used as starting values and the first-order factor variances were freely estimated. In addition, the variance of the second-order factor was fixed to one for identification in the EWC model. Considering this re-expressed nature, a EWC model, the original 14-item ESEM model, and the higher-order EWC model are supposed to produce identical model-fit indices as well as equivalent chi-square statistics, degrees of freedom, and estimated factor loadings, except the newly estimated second-order factor loadings, which were 0.81 (Factor 1, *p* < 0.001), 0.63 (Factor 2, *p* < 0.01), and 0.67 (Factor 3, *p* < 0.001). In sum, the ESEM results supported the structure of the three factors, representing the overall FOBism concept as a whole.

### 3.2. Reliability

The internal consistency reliability for the FOBism Scale scores was adequate, with a Cronbach’s alpha of 0.83 for the composite scores, 0.77 for the interpersonal subscale scores, 0.73 for the intrapersonal subscale scores, and 0.70 for the societal subscale scores. The ranges of these internal consistencies were all deemed “respectable” based on DeVellis’ study [48]. In other words, these items seemed to be working together to collectively assess the corresponding (sub)constructs of FOBism.

### 3.3. Construct Validity

FOBism scores were correlated with other study variables to establish construct validity (see Table 3). According to Cohen [49], correlation coefficients around 0.10 are considered weak associations, those around 0.30 moderate, and those above 0.50 strong. As expected, the FOBism scores were strongly and positively correlated with two related measures—the Colonial Mentality Scale–Within-Group Discrimination subscale (CMS-WGD; *r* = 0.38 to 0.52) and the Internalized Racism in Asian Americans Scale composite score (IRAAS; *r* = 0.34 to 0.58)—with the exception of FOBism Scale (FS)-Intrapersonal not being significantly correlated with CMS-WGD (*r* = 0.14), which is understandable, as the former focuses on self and the latter on others. The FOBism scores were moderately and positively correlated with Stress over the Perpetual Foreigner Stereotype scores (SPFS; *r* = 0.25 to 0.35) for those who came to the US after adolescence, and in contrast negatively correlated with the Foreigner Objectification Scale (FOS; *r* = −0.24 to −0.31) for those who were born in or came to the US prior to adolescence, with the exception of a nonsignificant correlation (*r* = −0.04) for FS-Intrapersonal. The FOBism scores also had moderately negative associations with the Collective Self-Esteem Private subscale scores (*r* = −0.23 to −0.33). The FOBism scores (composite, interpersonal, and intrapersonal) had moderate and negative associations with two aspects of the Asian American Multidimensional Acculturation Scale—connecting with Asian internationals (People-AI; *r* = −0.27 to −0.31) and identifying with their Asian Culture of Origin (Culture-As; *r* = −0.26 to −0.44). The FS-Societal subscale scores were positively associated with connecting with European Americans (people-EA; *r* = 0.26). The FS-Intrapersonal subscale scores were negatively associated with the fluency and frequent use of an Asian language (Lang-As; *r* = −0.28). However, there were no significant associations between FOBism scores with connecting with Asian Americans (People-AA), identifying with White Mainstream American Culture (Culture-Wh), and fluency and frequent use of English. The FOBism scores overall had a weak positive link with depression (*r* = −0.05 to 0.18). Lastly, as expected, the FOBism scores were not significantly correlated with social desirability. Overall, these associations with other study variables were in the expected direction, though some were nonsignificant. In other words, how the FOBism scores related to other associated variables provided support that the FOBism Scale measures what it was intended to measure.

### 3.4. Incremental Validity

We also conducted two sets of hierarchical regressions to examine the incremental validity of FOBism over and above two related measures, the Colonial Mentality Scale–Within-Group Discrimination subscale (CMS-WGP) and the Internalized Racism in Asian Americans Scale (IRAAS), in separately predicting four outcome variables—Collective Self-Esteem Scale (CSES-Pr), connection with Asian Internationals (People-AI), identifying with Asian culture of origin (Cult-As), and fluency in an Asian language (Lang-As).

A set of four separate regression analyses was conducted to examine the incremental validity of FOBism over and above CMS-WGP. In step 1, we entered CMS-WGP as a covariate. In step 2, we entered the three FOBism subscale scores as predictors (see Table 4). Hierarchical regression results partially supported the incremental validity of FOBism over and above CMS-WGP in predicting three of the four outcome variables—collective self-esteem (Δ*R*^2^ = 0.18, Δ*F*(1, 64) = 4.71, *p* < 0.01), connection with Asian Internationals (Δ*R*^2^ = 0.13, Δ*F*(1, 56) = 3.24, *p* < 0.05), and identifying with Asian culture of origin (Δ*R*^2^ = 0.18, Δ*F*(1, 64) = 4.91, *p* < 0.01).

Another set of four different regression analyses was conducted to examine the incremental validity of FOBism over and above IRAAS. In step 1, we entered IRAAS as a covariate. In step 2, we entered the three FOBism subscale scores as predictors (see Table 5). Hierarchical regression results partially supported the incremental validity of FOBism over and above IRAAS in predicting identifying with Asian culture of origin (Δ*R*^2^ = 0.13, Δ*F*(1, 68) = 4.01, *p* < 0.05) and fluency in an Asian language (Δ*R*^2^ = 0.13, Δ*F*(1, 68) = 3.25, *p* < 0.05). However, FOBism did not demonstrate incremental validity over and above IRAAS in predicting collective self-esteem and connection with Asian Internationals.

Overall, these results provide partial but adequate support for the incremental validity of FOBism (in particular, FS-Intrapersonal) above and beyond the CMS-WGP in predicting collective self-esteem as well as above and beyond both the CMS-WGP and IRAAS in predicting cultural orientation. In sum, the FOBism Scale appears to provide additional nuanced values beyond the existing scales of similar constructs, helping us understand how FOBism is related to how a person chooses which cultural values to embrace.

## 4. Discussion

Through this study, we created a 14-item scale that measures the construct of FOBism among Asians in the United States. The psychometric evaluation indicated an appropriately strong factor structure, construct validity, and internal consistency reliability, providing support for the FOBism Scale as a promising measure.

### 4.1. Construct Representation and Reliability

The final FOBism Scale items incorporated all three aspects of the construct based on David and colleagues’ [12] framework of interpersonal, intrapersonal, and societal orientations of internalized racism. Factor structure evaluation supported a second-order factor, indicating the use of a composite FOBism score along with three subscale scores. Moreover, the 14-item FOBism Scale demonstrated adequate reliability coefficients for composite and subscale scores (over 0.70 but less than 0.90), indicating strong internal consistency with minimal redundancy of items [48].

### 4.2. Construct Validity

FOBism scores correlated in expected directions with associated variables, providing evidence for convergent construct validity. For example, FOBism was positively correlated with two measures of internalized racism at a moderate to strong level. These significant associations with internalized racism scales not only support the construct validity of the FOBism Scale, but also align with themes of internalized racism expressed through defensive othering, disidentification, and disassociation found through qualitative interviews [50]. FOBism scores were also negatively correlated with a private sense of collective self-esteem, which aligns with past studies that found adhering to Asian values positively predicted collective self-esteem [51].

Moreover, FOBism scores in general negatively correlated with enculturation, or the process of learning one’s native culture (in this case, Asian culture) [52]. Note that this is different than acculturation, which is the process of learning a non-native culture (in this case, American mainstream culture). The intrapersonal aspect of FOBism was the only dimension negatively associated with an orientation toward Asian language. In contrast, FOBism scores minimally correlated with identifying with American mainstream people, culture, and language, with the exception of the societal aspect, which positively correlated with a connection to European Americans. This pattern of correlations with acculturation and enculturation measures highlights that the FOBism construct weighs more on having negative attitudes toward Asian culture (low enculturation) than being drawn toward American mainstream culture (high acculturation). Past studies have found different functions and impacts between enculturation and acculturation for different types of Asians in the United States. One study found enculturation to predict less social stress, whereas acculturation predicted more daily support [53]. Another study found acculturation and enculturation to serve as resources in slightly different ways between US-raised and non-US-raised Asian students [52]. Future studies may focus on FOBism and low enculturation to better understand its psychological impact.

The correlations between being perceived as foreigners and FOBism scores were unexpected, with varying results depending on at what stage of life participants came to the United States. Specifically, a positive correlation was found between FOBism and stress from being perceived as a perpetual foreigner for those who immigrated after the age of 13. In contrast, a negative correlation was observed with experiences of foreign objectification for individuals born in the US or those who arrived before adolescence. It was hypothesized that there would be a positive relationship between being perceived as a foreigner and FOBism, and this was somewhat confirmed. Individuals who came to the US after adolescence exhibited greater FOBism when perceived as a foreigner (e.g., “People criticize me for not speaking/writing English well”). However, for those who are likely well acculturated, being mistaken as not belonging (foreign objectification, e.g., “Had someone comment on or be surprised by your English language ability”) was actually linked with lower levels of FOBism. Although this result is based on the context of most participants experiencing very low levels of foreign objectification (*M* = 1.81, which is only slightly above the rating of 1 = never on a 4-point Likert scale), this unexpected finding calls for deeper and more nuanced investigation.

As for discriminant validity, FOBism scores were minimally associated with social desirability, as expected. This result indicates that participants’ responses to the FOBism Scale were fairly honest and not significantly impacted by an inclination to appear desirable. In other words, the FOBism Scale items were not worded in a way that would make participants worry about being perceived negatively for answering a certain way.

### 4.3. Incremental Validity

Lastly, FOBism scores demonstrated partial incremental validity in predicting private collective self-esteem along with the three dimensions of enculturation above and beyond colonial mentality and internalized racism. This demonstrates that FOBism, and particularly the intrapersonal subscale, addresses a unique aspect of assimilation-specific internalized racism and provides original insight into how Asians in the United States feel about their collective ethnic identity and their attitudes and behaviors toward Asian people, culture, and language. In other words, FOBism can be seen as an initial level of awareness that may eventually lead to a deeper recognition of feelings, such as shame, embarrassment, and inferiority. Therefore, acknowledging FOBism is an important first step in addressing the underlying beliefs or motivations related to colonial mentality and internalized racism, which are often more difficult to identify and confront.

### 4.4. Limiations and Future Directions

Despite the support of the FOBism Scale being a psychometrically sound measure, there are a few limitations of this study to note. First, the study was limited to the US context and the sample was not fully representative of the Asians living in the US, so the results are limited in generalizing to Asians living within the US and in different countries. Future research may incorporate Asians outside the US, such as those living in Asia, or even non-Asian ethnic groups living in the United States, as to better understand how FOBism exhibits across various groups and contexts, as FOBism is not limited to Asians. For example, “paisa” is a similar term to “FOB” used within the Latinx community to describe newly arrived immigrants [54]. Moreover, the relationships between FOBism and the study variables were correlational and could not explain directionality. There is also the potential to examine other variables, such as bicultural harmony, cultural intelligence, and cultural humility, in relation to FOBism. Lastly, the FOBism Scale measured behavioral and attitudinal aspects of the construct, but underlying motives were not fully assessed. In other words, some individuals may respond to the FOBism items simply due to a preference of cultural orientation rather than internalized racism.

### 4.5. Practical Implications

The FOBism Scale appears to be a promising new measure for research and change. Within the Asian community, FOBism is a pervasive yet often unspoken issue, which can lead to widespread discrimination and internalized negativity. The sensitive nature of confronting intra-community biases makes it hard to openly address. However, addressing the issue is critical in order to challenge and change the underlying attitudes contributing to this form of racism. Operationalizing FOB can allow for systematic research to shed light on how FOBism contributes to intra-ethnic othering and sub-ethnic identities [50].

This scale offers a tangible method for individuals to assess and understand how they feel about their Asian cultural identity. At the communal level, the scale highlights a particular form of internalized racism. Enhanced awareness of this type of discrimination enables Asians in the United States to recognize the aversive impact of FOBism on their community. Moreover, awareness may indirectly lead to more exploration and understanding of the systemic oppression and prevailing societal attitudes that have led to internalized racism [4]. For American society to dismantle these systems, they must first be aware of them.

Our ultimate aim is to mitigate FOBism, fostering a resurgence of pride in Asian cultural heritage and encouraging wider acceptance of bicultural identities. The labels of “FOB” and “Whitewashed” are a result of adapting to racial oppression in the form of being viewed as perpetual foreigners, and blame should not be placed within the Asian community. Instead, public recognition and understanding of the FOBism construct are vital for enabling Asians in the United States to heal from the damaging effects of societal pressures that have led to divisions and alienation within their own community. Ideally, Asians in the United States should not have to modulate their behavior to meet expectations of proper acculturation imposed by others, whether from within their community or the mainstream. Instead, they should feel free to express their culture, whether it be acculturated, enculturated, or some combination of both that suits them [1]. Ultimately, this study aspires to foster increased unity and harmony among Asians in the United States.

## Figures and Tables

**Table 1:** Fit indices for five exploratory structural equation models.

Item Number	*χ* ^2^	*d*.*f*.	CFI	TLI	RMSEA	90% C.I.
27	745.964 ***	273	0.898	0.868	0.077	0.070–0.083
18	298.691 ***	102	0.935	0.902	0.081	0.070–0.092
15 ^a^	182.410 ***	63	0.949	0.915	0.080	0.067–0.094
15 ^b^	126.610 ***	62	0.972	0.953	0.059	0.045–0.074
14 ^c^	110.787 ***	52	0.974	0.954	0.062	0.046–0.078

Note. *** *p* < 0.001. A correlated residual variance between items 10 and 18 was included in the model ^b^, but not in the model ^a^. ^c^ The fit indices of the 14-item exploratory structural equation model (ESEM) are the same as those of the ESEM-within-CFA model with a second-order factor. CFI = comparative fit index; TLI = Tucker–Lewis index; RMSEA = root mean square error of approximation; 90% C.I. = RMSEA 90% confidence interval.

**Table 2:** Factor loadings: exploratory structural equation model with 14 items.

Item	Fac 1	Fac 2	Fac 3
7. I find those who speak English without an Asian accent more attractive	**0.73**	0.24	−0.19
1. I prefer to work with Asians who are acculturated over those who are not	**0.72**	−0.26	−0.01
2. I perceive those who speak with an Asian accent as less intelligent	**0.70**	0.02	0.03
13. I would only date an Asian if they were acculturated	**0.62**	0.09	−0.07
10. I look down on Asians who dress like foreigners	**0.58**	−0.02	0.24
14. I feel embarrassed when I hear Asians speak poor English	**0.53**	−0.01	0.15
4. I avoid speaking an Asian language in public	−0.14	**0.68**	0.16
8. I avoid practicing Asian cultural traditions	0.14	**0.64**	0.06
5. I try to act mainstream American as much as possible	0.08	**0.65**	0.02
6. I feel uncomfortable wearing Asian traditional clothing	0.08	**0.63**	−0.03
11. Unassimilated Asians are why Asian Americans are viewed as perpetual foreigners	−0.04	0.00	**0.83**
12. Those who speak an Asian language in public make Asians look bad	0.04	0.20	**0.65**
3. Negative Asian stereotypes mostly stem from recent immigrants	0.12	−0.10	**0.63**
9. I think Asians in the US should use English as their primary language	0.05	0.11	**0.50**

Note. Statistically significant factor loadings according to the criterion from Norman and Streiner (2014) are in bold. Fac 1 = interpersonal, Fac 2 = intrapersonal, and Fac 3 = societal. The inter-factor correlations were as follows (*p* < 0.001 for all three): 0.52 (1 vs. 2), 0.56 (1 vs. 3), and 0.44 (2 vs. 3). The inter-factor correlations were as follows (*p* < 0.001 for all three): 0.52 (Fac 1 and Fac 2), 0.56 (Fac 1 and Fac 3), and 0.44 (Fac 2 and Fac 3).

**Table 3:** Intercorrelations between study variables.

	1	2	3	4	5	6	7	8	9	10	11	12	13	14	15	16	17	18
1. FOBism																		
2. FS-Inter	0.84 ***																	
3. FS-Intra	0.76 ***	0.42 ***																
4. FS-Societal	0.73 ***	0.45 ***	0.37 ***															
5. CMS-WGD	0.52 ***	0.57 ***	0.14	0.38 **														
6. IRAAS	0.58 ***	0.57 ***	0.34 **	0.48 ***	---													
7. SPFS	0.35 *	0.32 *	0.31 *	0.25	0.35	0.28												
8. FOS	−0.26 **	−0.24 *	−0.04	−0.31 **	−0.13	−0.03	---											
9. CSES-Pr	−0.33 ***	−0.30 ***	−0.24 **	−0.23 **	−0.18	−0.35 **	−0.06	0.06										
10. People-AI	−0.29 ***	−0.27 **	−0.31 ***	−0.08	−0.32 *	−0.18	0.06	0.09	0.11									
11. People-AA	0.09	0.04	0.17	0.00	0.02	−0.26 *	−0.05	−0.02	0.38 ***	−0.06								
12. People-EA	0.13	−0.06	0.17	0.26 **	0.13	0.18	0.16	−0.11	−0.01	0.06	0.17							
13. Cult-As	−0.34 ***	−0.26 **	−0.44 ***	−0.10	−0.17	−0.39 ***	−0.18	0.16	0.35 ***	0.49 ***	−0.01	−0.05						
14. Cult-Wh	0.13	0.05	0.12	0.15	0.12	0.12	-0.05	−0.23 *	0.10	0.11	0.26 **	0.46 ***	0.01					
15. Lang-As	−0.15	−0.08	−0.28 ***	0.00	−0.04	0.04	−0.04	0.29 **	−0.16	0.56 ***	−0.39 ***	−0.08	0.48 ***	−0.11				
16. Lang-Eng	−0.01	0.00	0.07	−0.12	−0.01	−0.18	−0.19	−0.17	0.22 **	−0.31 ***	0.48 ***	0.16	−0.19 *	0.22 **	−0.44 ***			
17. DASS-Dep	0.17 *	0.15	0.18 *	0.05	0.07	0.17	0.22	0.05	−0.26 **	−0.15	−0.07	−0.04	−0.25 **	−0.09	−0.17 *	0.01		
18. SocDes	0.04	0.15	−0.08	−0.01	0.05	0.17	0.23	0.12	−0.11	0.06	−0.10	−0.14	0.01	−0.22 *	0.08	−0.08	0.25 **	
Mean	2.31	2.39	2.45	2.05	2.05	1.81	2.16	1.84	6.05	4.22	5.01	3.93	5.02	3.38	3.72	5.73	1.80	2.38
SD	0.67	0.81	0.98	0.82	0.70	0.64	0.77	0.71	1.01	0.97	0.90	0.92	0.97	1.16	1.62	0.51	0.70	0.64
N	295	295	295	294	69	74	46	102	144	129	128	128	144	143	144	144	144	143

Note. * *p* < 0.05, ** *p* < 0.01, and *** *p* < 0.001. CMS-WGD = Colonial Mentality Scale–Within-Group Discrimination; IRAAS = Internalized Racism in Asian Americans Scale; SPFS = Stress over the Perpetual Foreigner Stereotype; FOS = Foreigner Objectification Scale; CSES-Pr = Collective Self-Esteem Scale–Private; People-AI = connection with Asian Internationals; People-AA = connection with Asian Americans; People-EA = connection with European Americans; Cult-As = identifying with Asian culture of origin; Cult-Wh = identifying with White mainstream American culture; Lang-As = fluency in an Asian language; Lang-Eng = fluency in English; DASS-Dep = depression; SocDes = social desirability.

**Table 4:** Hierarchical regressions for incremental validity over and above colonial mentality.

	*B*	*S.E.*	*β*	Δ*R*^2^	Δ*F*	*df*s
***Collective Self-Esteem Scale–Private***						
Step 1				0.03	2.23	1, 67
CMS–Within-Group Discrimination	−0.27	0.18	−0.18			
Step 2				0.18 **	4.71	1, 64
CMS–Within-Group Discrimination	−0.04	0.21	−0.02			
FS-Interpersonal	−0.20	0.17	−0.18			
FS-Intrapersonal	−0.40	0.14	−0.34 **			
FS-Societal	−0.02	0.14	−0.02			
***Connection with Asian Internationals***						
Step 1				0.10 *	6.76	1, 59
CMS–Within-Group Discrimination	−0.45	0.17	−0.32 *			
Step 2				0.13 *	3.24	1, 56
CMS–Within-Group Discrimination	−0.55	0.22	−0.40 *			
FS-Interpersonal	0.08	0.17	0.08			
FS-Intrapersonal	−0.42	0.15	−0.37 **			
FS-Societal	0.18	0.13	0.17			
***Identifying with Asian Culture of Origin***						
Step 1				0.03	1.90	1, 67
CMS–Within-Group Discrimination	−0.25	0.18	−0.17			
Step 2				0.18 **	4.91	1, 64
CMS–Within-Group Discrimination	−0.22	0.21	−0.14			
FS-Interpersonal	−0.02	0.17	−0.02			
FS-Intrapersonal	−0.52	0.15	−0.43 ***			
FS-Societal	0.13	0.14	0.12			
***Fluency in an Asian language***						
Step 1				0.00	0.09	1, 67
CMS–Within-Group Discrimination	−0.09	0.29	−0.04			
Step 2				0.05	1.13	1, 64
CMS–Within-Group Discrimination	−0.19	0.36	−0.08			
FS-Interpersonal	0.07	0.29	0.04			
FS-Intrapersonal	−0.41	0.25	−0.22			
FS-Societal	0.23	0.25	0.13			

Note. * *p* < 0.05, ** *p* < 0.01, and *** *p* < 0.001.

**Table 5:** Hierarchical regressions for incremental validity over and above internalized racism.

	*B*	*S.E.*	*β*	Δ*R*^2^	Δ*F*	*df*s
***Collective Self-Esteem Scale–Private***						
Step 1				0.08 *	6.03	1, 71
Internalized Racism in Asian Americans	−0.42	0.17	−0.28 *			
Step 2				0.03	0.83	1, 68
Internalized Racism in Asian Americans	−0.33	0.22	−0.22			
FS-Interpersonal	−0.02	0.23	−0.01			
FS-Intrapersonal	0.13	0.13	0.13			
FS-Societal	−0.24	0.17	−0.20			
***Connection with Asian Internationals***						
Step 1				0.04	2.66	1, 65
Internalized Racism in Asian Americans	−0.31	0.19	−0.20			
Step 2				0.09	2.24	1, 62
Internalized Racism in Asian Americans	−0.02	0.24	−0.01			
FS-Interpersonal	−0.50	0.26	−0.32			
FS-Intrapersonal	−0.20	0.14	−0.19			
FS-Societal	0.17	0.19	0.14			
***Identifying with Asian Culture of Origin***						
Step 1				0.15 ***	12.02	1, 71
Internalized Racism in Asian Americans	−0.55	0.16	−0.38 ***			
Step 2				0.13 *	4.01	1, 68
Internalized Racism in Asian Americans	−0.43	0.19	−0.30 *			
FS-Interpersonal	−0.15	0.20	−0.11			
FS-Intrapersonal	−0.36	0.11	−0.38 **			
FS-Societal	0.25	0.15	0.22			
***Fluency in an Asian language***						
Step 1				0.00	0.00	1, 71
Internalized Racism in Asian Americans	−0.02	0.30	−0.01			
Step 2				0.13 *	3.25	1, 68
Internalized Racism in Asian Americans	0.12	0.36	0.05			
FS-Interpersonal	0.04	0.37	0.02			
FS-Intrapersonal	−0.66	0.21	−0.40 **			
FS-Societal	0.29	0.29	0.15			

Note. * *p* < 0.05, ** *p* < 0.01, and *** *p* < 0.001.

## Data Availability

Data are available upon request.
